# Absorption Characteristics of Combination Medication of Realgar and* Indigo Naturalis*:* In Vitro* Transport across MDCK-MDR1 Cells and* In Vivo* Pharmacokinetics in Mice after Oral Administration

**DOI:** 10.1155/2018/6493630

**Published:** 2018-09-06

**Authors:** Miao Zhang, Lin Guo, Long-Fei Lin, Chang-Hai Qu, Xing-Bin Yin, Shi-Lin Luo, Xin Zhang, Hai-Ying Zhang, Xiao Liang, Jun Guan, Jian Ni

**Affiliations:** ^1^Xiyuan Hospital, China Academy of Chinese Medical Sciences and Beijing Key Lab of TCM Pharmacology, Beijing 100091, China; ^2^School of Chinese Materia Medica, Beijing University of Chinese Medicine, Beijing 100029, China; ^3^Institute Chinese Materia Medica, China Academy of Chinese Medical Sciences, Beijing 100700, China; ^4^Shanghai Binuo Medical Instrument Co., Ltd., Shanghai 201414, China; ^5^Beijing Research Institute of Chinese Medicine, Beijing University of Chinese Medicine, Beijing 100029, China

## Abstract

Realgar and* indigo naturalis* are clinically combined to treat varieties of leukemia. Exploring the drug-drug interactions might be beneficial to find active substances and develop new targeted drugs. This study aimed at exploring the change of arsenic concentration in mice and across MDCK-MDR1 cells and the cytotoxicity on K562 cells when realgar and* indigo naturalis* were combined. In the presence or absence of* indigo naturalis*, pharmacokinetics and cell-based permeability assays were used to evaluate the change of arsenic concentration, and K562 cell line was applied to evaluate the change of cytotoxicity. The drug concentration-time profiles exhibited that the combination medication group generated higher AUC, t_half_, and longer MRT for arsenic, compared with the single administration of realgar. The apparent permeability coefficients (P_app_) of bidirectional transport in MDCK-MDR1 cell permeability experiments showed that arsenic permeability obviously went up when* indigo naturalis* was incubated together. The combination medication significantly decreased the cell viability of K562 cells when both the concentration of realgar and the concentration of* indigo naturalis* were nontoxic. The pharmacokinetic research, the MDCK-MDR1 based permeability study, and the K562 cytotoxicity study were united together to verify the combination medication of realgar and* indigo naturalis* enhanced the absorption and the permeability across cells for arsenic and effectively inhibited the proliferation of K562 cell line. The molecular binding of As_4_S_4_ and indirubin was analyzed by computational study. It is predicted that the formation of the complex [As_4_S_4_^…^Indirubin] involves noncovalent interaction that changes the concentration of arsenic.

## 1. Introduction

Arsenicals such as As_2_O_3_ and realgar (As_4_S_4_) have been used to treat hematological malignancy widely in both Eastern and Western world [1, 2]. One of the most famous arsenicals is the ATO injection (As_2_O_3_), Trisenox®, which shows remarkable effect against APL after being approved by FDA [3]. Another famous arsenical is realgar, which has been proved to be as effective as arsenic trioxide with less irritation according to the clinical trials and treatments [4, 5]. Nowadays, oral administration of realgar is utilized widely to treat APL and MDS in clinical therapeutics [5, 6]. In fact, arsenicals have long been used in TCM to treat a variety of diseases, including syphilis, malaria, skin problems, and several other parasitic infections for more than 1500 years [7]. TCM practitioners for millennia have accumulated some therapeutic experiences prescribing a combination of arsenicals with several other Chinese herbal medicines or mineral medicines [8, 9]. Realgar and* indigo naturalis* are a common combination regimen therapeutically used in the treatment of different types of leukemia [8–10]. Leukemia is a group of malignant clonal diseases of hematopoietic stem cells [11]. The ineffective hematopoiesis results in the decrease of normal blood cells [12]. There are four main types of leukemia, acute lymphoblastic leukemia (ALL), acute myeloid leukemia (AML), chronic lymphocytic leukemia (CLL), and chronic myeloid leukemia (CML), as well as a number of less common types [5, 6, 13, 14].

Realgar, a mineral arsenical drug, contains more than 90% As_4_S_4_ and is regarded as the principle medicine to provide arsenic [15]. Indirubin, a 3,2′-bisindole isomer of indigo, was a stable and active ingredient of* indigo naturalis* [16, 17]. Zhou et al. (1981) clinically used realgar and* indigo naturalis* in a formula to treat patients with CML [18]. Subsequently, realgar-*indigo naturalis* formula (RIF) which contains realgar,* indigo naturalis*, and* salvia miltiorrhiza* was found effective against APL in some clinical trials [8]. An observation in their work was that the combination therapy of their main active ingredients, As_4_S_4_, indirubin, and tanshinone IIA can increase the cellular uptake of arsenic in NB4 cells through significantly inducing upregulation of AQP9 which is a transmembrane protein playing an essential role in arsenic uptake and determining cellular arsenic sensitivity, compared with As_4_S_4_ treatment alone. The upregulation of AQP9 was detected at 24 h after combination treatment, but some other events or interactions were not addressed in their paper that might also lead to the enhancement of arsenic action at the earlier stage. Here, the pharmacokinetic study in mice whole blood was monitored between 0 and 24h after medication to compare realgar pharmacokinetic parameters with or without* indigo naturalis*. In order to further study the effects of* indigo naturalis* on the penetration of arsenic, the MDCK-MDR1 based permeability assay was used to analyze the arsenic transport across cells. For proving the combination effect of realgar and* indigo naturalis* on treating CML, K562 cell line was incubated to evaluate the cytotoxicity.

## 2. Methods

### 2.1. Materials and Reagents

Realgar was provided by Department of Hematology, Xiyuan Hospital, China Academy of Chinese Medical Sciences (Beijing, China).* Indigo naturalis* was purchased from Beijing Bencaofangyuan Co. Ltd. (Beijing, China). Standard solutions of As were prepared by using the stock solution which was purchased from National Institute of Metrology, China. Nitric acid (65%-68% v/v, guarantee reagent) and hydrogen peroxide (30% v/v, guarantee reagent) were purchased from Beijing Chemical Works. Ultrapure water was produced by Milli-Q water (Millipore, Bedford, MA, USA). Dulbecco's modified eagle medium (DMEM), RPMI 1640 culture medium, fetal bovine serum, trypsin/EDTA, penicillin, and streptomycin were purchased from Gibco-Life Technologies (USA). Hanks balanced salt mixture, phosphate buffer solution (PBS), and dimethyl sulfoxide (DMSO) were purchased from Solarbio (Beijing, China). 3-(4,5-Dimethylthiazol-2-yl)-2,5-diphenyltetrazolium bromide (MTT) was obtained from Biotopped Life Sciences (Beijing, China). Polyester transwells (3460) were purchased from Costar (Cambridge, MA). Male ICR mice were purchased from Beijing Vital River Laboratory Animal Technology Co., Ltd. (Beijing, China) and were housed in a temperature and humidity controlled environment and maintained in a 12-hour light/dark cycle for three days with free access to food and water. Electrical resistor (Millicell® ERS-2) was purchased from Millipore (USA). The human CML cell line K562 was obtained from the Chinese Academy of Medical Sciences & Peking Union Medical College (Beijing, China).

### 2.2. Apparatus

An Agilent 7700 inductively coupled plasma mass spectrometer (ICP-MS) (Agilent Technologies, Palo Alto, CA, USA) was used to detect and quantify arsenic. 1.0 *μ*g/L of mass spectrometer turned liquid and 1.0 *μ*g/L of online internal standard solution were used to optimize the conditions of ICP-MS. The radio frequency power was set as 1500 W; the plasma gas flow rate was 15.0 L/min; the auxiliary flow rate was 1.0 L/min; the carrier gas flow was 1.0 L/min; the sampling deepness was 7.5 mm; the aperture of sampling cone was 1.0 mm; the aperture of skimmer cone was 0.4 mm; the temperature in spray chamber was 2°C; the peristaltic pump speed was 0.1 rps. The analysis room vacuum degree was ranging from 1 × 10^−5^ Torr to 2 × 10^−5^ Torr. The measurement mode adopted peak jumping type. The data collection repetition times were set as three times, sampling points for each peak were three. The increasing rate of test solution was 1.0 mL/min.

Microwave digestion system (MARS 6 MARSXpress, CEM Corporation, Matthews, NC, USA) was used to digest samples. Samples were digested in seven steps shown in Table 1.

### 2.3. Oral Administration Methods

Researchers found that rats had a longer retention time of arsenic in their blood than that in humans mainly because of the retention in RBCs. Hemoglobin in RBCs has been regarded as a target protein of arsenic. Arsenic can significantly accumulate in rat RBCs because of the binding of the reactive arsenic metabolite, dimethylarsinous acid (DMA^III^), to a highly reactive cysteine residue in rat hemoglobin (cys-13 in the alpha chain) [19]. Therefore, it is suggested that rats are not appropriate for model animals of arsenic pharmacokinetic studies. All animal experimental procedures were followed by the National Institutes of Health Guide for the Care and Use of Laboratory Animals were approved by the committee of Animal Care and Utilities.

#### 2.3.1. Pharmacokinetic Method

Realgar (R) and the combination of realgar and* indigo naturalis* (RI) were both mixed in 0.5% CMC-Na solution. Mice were orally dosed with R (606 mg/kg) and RI (606 mg/kg of R, 1212 mg/kg of I), respectively. Whole blood samples (about 0.1 ml) were drawn from ocular fundus veins of mice at 0, 0.083, 0.25, 0.5, 0.75, 1, 2, 4, 8, 12, and 24 h after dosing and treated with nitric acid and hydrogen peroxide for microwave digestion. Using Ge and Rh as internal standard, samples were detected by ICP-MS to determine the concentration of arsenic. The pharmacokinetic parameters of As were calculated by Kinetica 4.4 software (Thermo Scientific, USA).

#### 2.3.2. Multiple Oral Administration Method

Realgar (R) and the combination of realgar and* indigo naturalis* (RI) were both mixed in 0.5% CMC-Na solution. Mice were orally dosed with R (606 mg/kg) and RI (606 mg/kg of R, 1212 mg/kg of I), respectively, for seven days. Whole blood samples (about 0.1 ml) were drawn from ocular fundus veins of mice after the administration for seven days and treated with nitric acid and hydrogen peroxide for microwave digestion. Using Ge and Rh as internal standard, samples were detected by ICP-MS to determine the concentration of arsenic.

#### 2.3.3. Microwave Digestion for Blood Samples

Under the setting programs (shown in Table 1), whole blood samples were digested by microwave digestion system with 8 mL of nitric acid and 2 mL of hydrogen peroxide in Teflon vessels, followed by electric heating plate to volatilize spare liquid. After being cooled, the remaining droplets were poured out from Teflon vessels and diluted with ultrapure water to certain volumes.

### 2.4. Transmembrane Transport across MDCK-MDR1 Cells

#### 2.4.1. Cell Culture

MDCK-MDR1 cells (1 × 10^6^ cells/mL) were cultured in DMEM supplemented with 10% FBS, 1% penicillin, and streptomycin at 37°C in a tissue culture incubator with 5% CO_2_ and 95% air for 2-3 days to reach 80% confluence. Cells (4× 10^5^ cells/mL, 0.5 mL) were then seeded onto transwells and incubated for 4-5 days to reach cell monolayers. The integrity of cell monolayers was assessed by measuring the transepithelial electrical resistance (TEER). 250-350 Ω·cm^−2^ of TEER presented cells connected tightly and cell monolayer was integrated. The medium was replaced with fresh medium every day.

#### 2.4.2. Bidirectional Permeability Measurements

Medium from all apical inserts and basolateral wells was replaced with HBSS solution for 30 min at 37°C in incubator. Compound permeability was assayed by adding drugs to an apical insert for the apical to basolateral (A to B) transport and to a basolateral well for the basolateral to apical (B to A) transport. For A to B transport, 0.5 mL of drug solution was added in A side and 1.5 mL of HBSS in B side to measure A-B transporter, and 600 *μ*L of uptake solution samples was collected from the B side and diluted with ultrapure water. For B to A transport, 1.5 mL of drug solution was added in B side and 0.5 mL of HBSS in A side to measure B-A transporter, and 200 *μ*L of efflux solution was collected from the A side and diluted with ultrapure water.

Realgar (R) was suspended in PBS;* indigo naturalis* (I) was suspended in DMSO. The cells were treated with R (35 *μ*g/mL of R) and RI (35 *μ*g/mL of R and 70 *μ*g/mL of I), respectively. The proportion of realgar and* indigo naturalis* was followed with the proportion in pharmacokinetic study. The treated cells were then returned to the incubator at 37°C. The samples were collected at 30 min, 60 min, 90 min, 120 min, 150 min, and 180 min from both apical inserts and basolateral wells. Using Ge and Rh as internal standard, samples were detected by ICP-MS to determine the concentration of arsenic.

### 2.5. Cytotoxic Effects on K562 Cell Line

K562 cells were cultured in RPMI 1640 medium supplemented with 10% FBS and 1% antibiotics (penicillin/streptomycin) in a humidified 37°C chamber with 5% CO_2_ [20]. The cytotoxic effects of realgar (R),* indigo naturalis* (I), and realgar and* indigo naturalis *(RI) on K562 cells were evaluated by MTT assays. For MTT assays, K562 cells were seeded in 96-well flat-bottom microtiter plates at a density of 1 × 10^5^ cells/mL with 100 *μ*L of cell solution.

Realgar (R) was suspended in PBS;* indigo naturalis* (I) was suspended in DMSO. K562 cells were, respectively, treated with various concentrations of R (0, 3.5, 7, 14, and 28 *μ*g/mL), I (0, 7, 14, 28, and 56 *μ*g/mL), and RI (0, 3.5, 7, 14, and 28 *μ*g/mL of R and 0, 7, 14, 28, and 56 *μ*g/mL of I). The proportion of realgar and* indigo naturalis* was followed with the proportion in pharmacokinetic study. PBS and DMSO were used as the vehicle control. The treated cells were incubated for 24 h in a humidified 37°C chamber with 5% CO_2_. Then the medicated cells were treated with MTT working solution (0.5 mg/mL) for 3-4 h at 37°C. The plates were centrifuged at 800 g for 5 min, and the supernatant was subsequently removed from all the wells. Then, 150 mL of DMSO was added to dissolve the formazan crystals precipitated at the bottom. The absorbance of the formazan solution was measured at 570 nm by a microplate reader.

### 2.6. Apoptosis Assay

K562 cells were cultured in RPMI 1640 medium supplemented with 10% FBS and 1% antibiotics (penicillin/streptomycin) in a humidified 37°C chamber with 5% CO_2_. Realgar (R) was suspended in PBS,* indigo naturalis* (I) was suspended in DMSO. K562 cells were seeded in six-well plates at a density of 4 × 10^5^ cells per well. The cells were, respectively, incubated with R (0, 28 *μ*g/mL) and RI (0, 28 *μ*g/mL of R and 0, 56 *μ*g/mL of I). The treated cells were incubated in a humidified 37°C chamber with 5% CO_2_ for 24 h. PBS and DMSO were used as the vehicle control. The apoptotic K562 cells were resuspended in 295 *μ*L of binding buffer and stained with 5 *μ*L of Annexin V-FITC and 10 *μ*L of propidium iodide (PI). Next, the cells were incubated for 20 min in the dark. The viable, apoptotic, and necrotic cell numbers were determined by flow cytometry.

### 2.7. ICP-MS Analysis

The method assay was validated according to FDA guidelines (US Food and Drug Administration, 2001). Calibration samples (1, 2, 5, 10, 20, 50, and 100 *μ*g/L) and the quality control (QC) samples (5, 50, and 80 *μ*g/L) were prepared by spiking blank whole blood and serial diluting to the desired concentration. The linearity of the calibration curve was determined by plotting the peak area ratios of analytes to IS (Y) versus the spiked concentrations of analytes (X), and it was considered valid only if r^2^ >0.99. The specificity of this method was established by quantifying blank whole blood samples from randomly selected ICR male mice (n=10). Lowest limits of quantitation (LLOQ) was expressed as 3-fold of ratio of the signal-to-noise ratio. The LLOQ was defined as the lowest concentration in the calibration curve with the acceptable precision (RSD<20%) and accuracy (within 100±20%). The precision and accuracy of QC replicates (5 *μ*g/L, 50 *μ*g/L, and 80 *μ*g/L) were detected on the same day, and the precision and accuracy for three batches of QC replicates were detected on three consecutive validation days; the precisions should not exceed 15%, and the accuracies were required to be within 100±20%. The stability for QC replicates was investigated under the following three conditions: QC replicates were, respectively, stored at 4°C for 24 h, stored at −20°C for one month, and stored at three freeze-thaw cycles, and they were considered stable when 85-115% of the initial concentrations were obtained.

## 3. Results

### 3.1. Method Validation

The calibration curve was y=0.0065 x + 0.0041, and the correlation coefficient (r^2^) was 0.9999. The calibration curve showed good linearity in the range of 1-100 *μ*g/L for arsenic. The quantification of arsenic concentration in blank blood was detected with 10 mice. It exhibited significant interference from endogenous substance. Arsenic was an element originally existing in mice blood, and the total arsenic content in individual mice blood was different. In order to keep the consistency of arsenic content in blank blood which was used in the following method assays, 20 ml of blank blood was collected from mice and mixed together for the subsequent use. The LLOQ for total arsenic was 1 *μ*g/L, which was sensitive enough for the pharmacokinetic study of total arsenic in mice blood. The RSD of LLOQ was 2.11% and the accuracy of LLOQ was 102.40%. The intra- and interday precision and accuracy values of this method were investigated by analyzing QC samples (5, 50, and 80 *μ*g/L). The intraday RSD was below 3.0%, and the interday RSD was below 4.0%. The intraday accuracy ranged from 87.0% to 104.0%, and the interday accuracy ranged from 87.0% to 108%. This method was proved to be highly accurate and precise. The stability of total arsenic under various storage conditions was tested. Stability studies showed that total arsenic in samples was stable at the condition of 4°C for 24 h and the condition of three freeze-thaw cycles. However, the concentrations of total arsenic significantly decreased after being stored at −20°C for one month.

### 3.2. Combination Medication of Realgar and* Indigo Naturalis* Enhances Arsenic Absorption in Mice Whole Blood

This study focused on the changes of arsenic concentration in whole blood after the administration of realgar with or without* indigo naturalis*.

**Figure 1:**
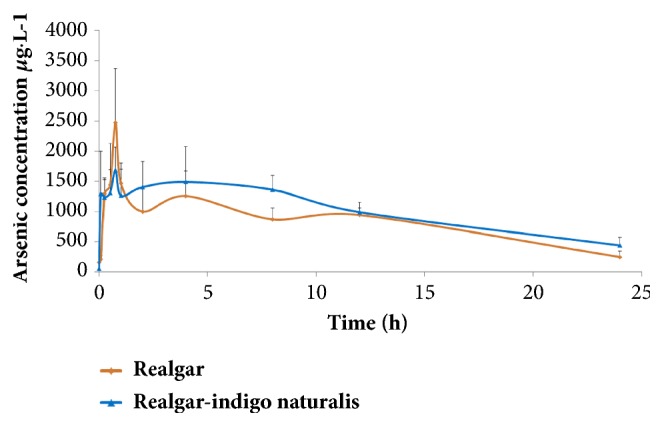
Mean (±SD) whole blood concentration of arsenic versus time profiles after oral administration of realgar and combined administration of realgar and* indigo naturalis*.

The PK parameters of R can be found mostly lower than that of RI. Arsenic whole blood concentration after the administration of R reached the maximum at 45 min with an average C_max_ of 2472.41 *μ*g/L. The area under the curve AUC_(0-24)_ was 18992.00 (h *μ*g/L), AUC_(0-∞)_ was 22039.2 (h *μ*g/L), half-life was 8.58 h, and mean residence time (MRT) was 12.97 h, whereas arsenic whole blood concentration after the combination medication reached the maximum at 45 min with an average C_max_ of 1681.32 *μ*g/L. AUC_(0-24)_ grew to 24057.60 (h *μ*g/L), AUC_(0-∞)_ changed to 30340.00 (h *μ*g/L), half-life prolonged to 9.91 h, and MRT extended to 15.57 h. In general, the addition of* indigo naturalis* increased AUC, half-life, and MRT in spite of decreasing C_max_. AUC_(0-∞)_ reflects the actual body exposure to drug after administration of a dose of the drug from time zero to infinite time, corresponding to the total amount of the dose absorbed in the systematic circulation. The half-life t_half_ of the drug is the time to eliminate 50% of the drug blood concentration in the body. Increased t_half_ implied the elimination rate became slow. To discuss MRT, it is essential to change the idea to the residence time of individual molecules in the body. Each molecule spends a different period of time in the body, with some molecules lasting a very short amount of time and others lasting longer. MRT reflects overall behaviors of individuals. Therefore, the higher PK parameters of the AUC, t_half_, and MRT presented after arsenic medication, the bigger amount of arsenic was absorbed by the bloodstream, the longer time was spent in the systematic circulation, and the slower decline in the blood arsenic concentration. The pharmacokinetic profile (mean whole blood concentration of arsenic versus time (n=6)) was shown in Figure 1, and the main pharmacokinetic parameters derived from these data are summarized in Table 2.

After consecutive oral administration of R and RI in mice for seven days, the arsenic concentration of R can be found lower than that of RI. Arsenic whole blood concentration after the accumulative administration of R reached the 3361.34 *μ*g/L. Arsenic whole blood concentration after the accumulative administration of RI reached 4239.15 *μ*g/L.

### 3.3. Combination Medication of Realgar and* Indigo Naturalis* Increases Arsenic Transport on MDCK-MDR1 Cell Line

The apparent permeability coefficients (P_app_) were calculated according to (1): P_app_= (dQ /dt) / AC, where dQ /dt is the apparent appearance rate of arsenic in the receiver side calculated using linear regression of amounts in the receiver chamber versus time, A is the surface area of the polyester membrane of Transwell (cm^2^), and C is the arsenic concentration incubated in donor side. The efflux ratio (ER) was calculated according to (2):

ER=Papp (B-A)/Papp (A-B).

**Figure 2:**
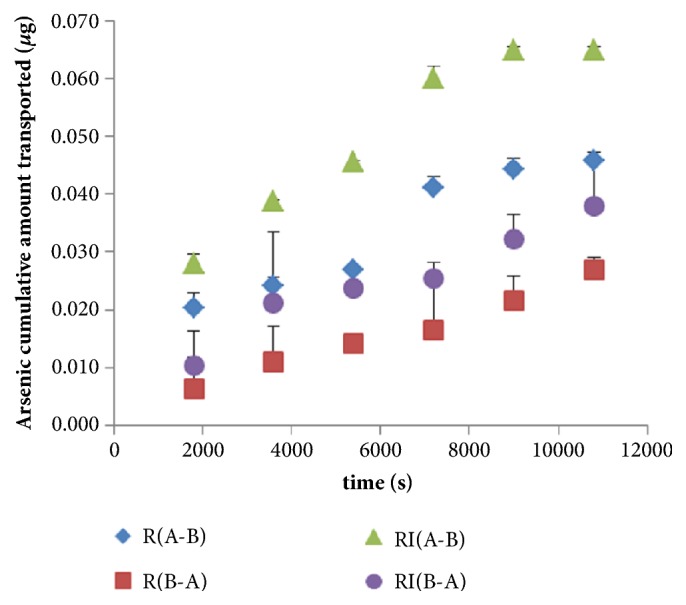
Cumulative amount of transported arsenic in R and RI across MDCK-MDR1 cells.

**Figure 3:**
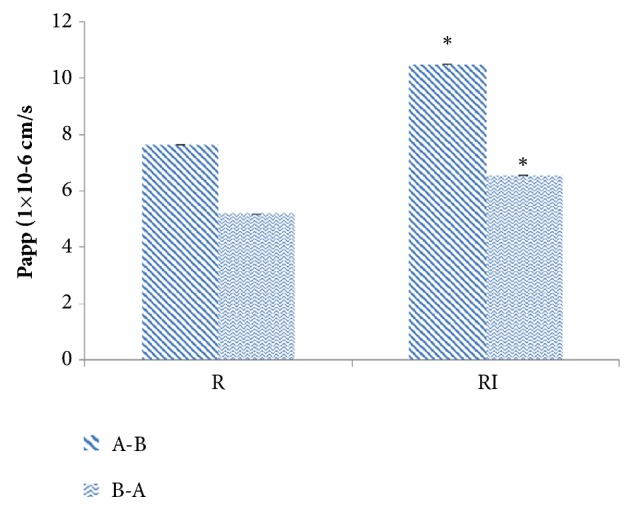
Apparent permeability coefficients of transported arsenic in R and RI across MDCK-MDR1 cells. Data are represented as the mean ± SD from three independent experiments (*∗*p < 0.05 versus control).

In order to study the effects of* indigo naturalis* on arsenic transport, the permeability of arsenic was assessed in the presence or absence of* indigo naturalis* across MDCK-MDR1. The cumulative amount and Papp of transported arsenic were calculated. Results were expressed as mean±SD, n=3, shown in Figure 2. The apparent permeability coefficients (×10^−6^ cm/s) of realgar alone and the combination were listed in Table 3 and Figure 3. All of the apparent permeability coefficients were greater than 2 × 10^−6^ and lower than 20 × 10^−6^ in MDCK-MDR1 cells, which proved that arsenic should be classified to medium permeability drugs absorbed by small intestine [21]. In addition, the efflux ratios (ER) of arsenic in R and RI groups were between 0.5 and 2 in MDCK-MDR1 cells, which indicated that arsenic entered into cells through nonactive transport [22]. Moreover, the Papp A-B or Papp B-A values increased when cells were treated with realgar and* indigo naturalis*, compared to the treatment with realgar alone. Therefore, the results manifested that* indigo naturalis* could enhance the arsenic penetration amount and permeability rate. The MDCK-MDR1 based permeability results were consistent with the pharmacokinetic study. Drug-drug interaction between realgar and* indigo naturalis* occurred mainly in the absorption process.

### 3.4. Combination Medication of Realgar and* Indigo Naturalis* Increases the Cytotoxic Effect of K562 Cells

**Figure 4:**
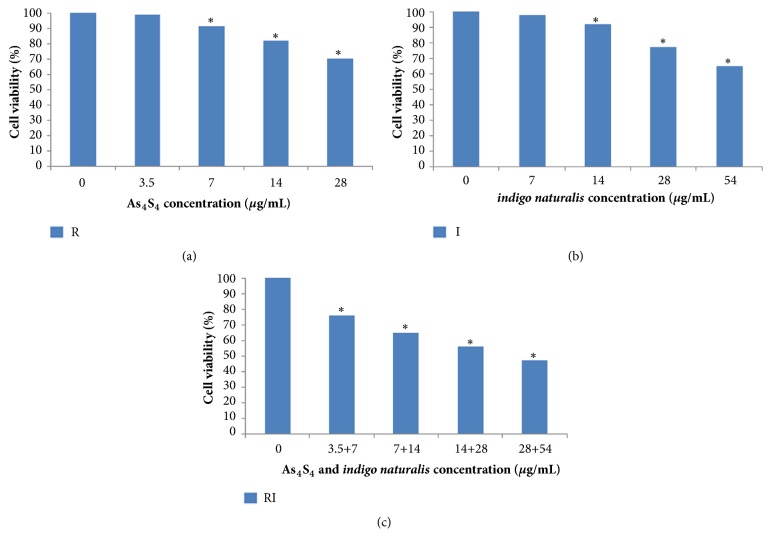
Effects of realgar (R),* indigo naturalis* (I), and realgar and* indigo naturalis* (RI) on K562 cell viability. (a) The cell viability of K562 after treatment with various concentrations of R (0, 3.5, 7, 14, and 28 *μ*g/mL) for 24 h. (b) The cell viability of K562 after treatment with various concentrations of I (0, 7, 14, 28, and 56 *μ*g/mL) for 24 h. (c) The cell viability of K562 after treatment with various concentrations of RI (0, 3.5, 7, 14, and 28 *μ*g/mL of R and 0, 7, 14, 28, and 56 *μ*g/mL of I) for 24 h. Data are represented as the mean ± SD from three independent experiments (*∗*p < 0.05 versus control).

In order to study the effects of combination therapy on K562 cell line, K562 cells were exposed to R, I, and RI. MTT assays were used to measure the cell viability of K562 cells after 24 h of drug treatment. Results were expressed as mean±SD, n=3, shown in Figure 4. The results showed that the cell viability effectively decreased when R and I were combined, while the cell viability was not decreased after the single medication at the same single concentration. In general, the combined medication group showed strong cytotoxicity and significantly decreased K562 cells proliferation and viability compared to that of the vehicle controls. (shown in Figure 4).

### 3.5. Combination Medication of Realgar and* Indigo Naturalis* Increases the Apoptotic Effect of K562 Cells

**Figure 5:**
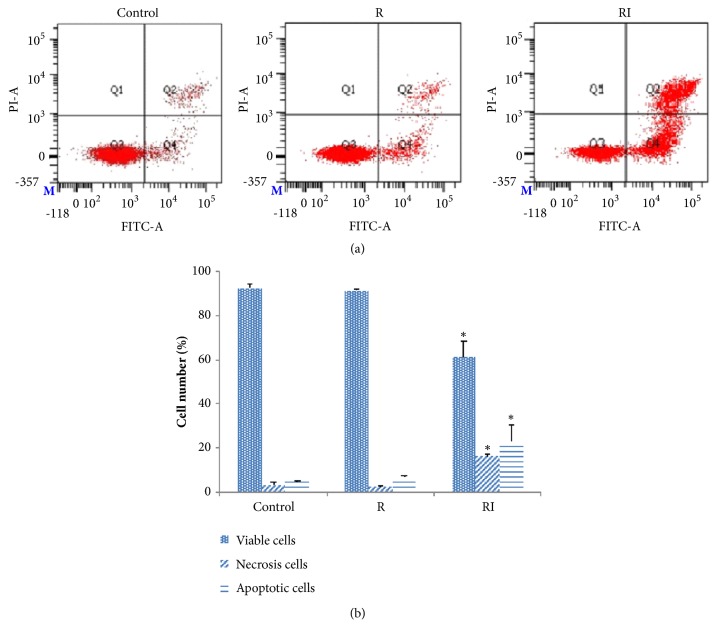
Effects of R and RI on apoptosis in K562 cells. (a) Quantification of viable, apoptotic and necrotic cells by flow cytometry with FITC-Annexin V/PI double staining after treatment with R (0, 28 *μ*g/mL) and RI (0, 28 *μ*g/mL of R and 0, 56 *μ*g/mL of I) for 24 h. (b) Percentages of viable, apoptotic and necrotic cells after treatment with R (0, 3.5 *μ*M) and RI (0, 3.5 *μ*M of R and 0, 7 *μ*M of I) for 24 h. Data are represented as the mean ± SD from three independent experiments (*∗*p < 0.05 versus control).

After K562 cells were treated with R and RI for 24 h, the proportion of viable cells was substantially reduced from 91.50% ± 2.51% to 56.30% ± 7.83%. In addition, the proportion of early apoptotic cells increased from 6.20% ± 0.51% to 28.60% ± 11.58%, along with a strong increase in necrotic and late apoptotic cells from 2.40% ± 1.44% to 15.10% ± 2.98%. These data indicated that R and RI reduce the viability of K562 cells through inducing K562 cell apoptosis (shown in Figure 5).

## 4. Discussions

Both of the oral administration study and the cell-based permeability study manifested a phenomenon that the additive* indigo naturalis* facilitated the arsenic absorption. And the cytotoxicity study indicated that the combination medication efficiently inhibited the proliferation of K562 cell line when neither the concentration of realgar nor the concentration of* indigo naturalis* was lethal. In order to further discuss the reason why* indigo naturalis *enhanced the arsenic absorption and permeability, we conducted the inference with the help of computational methods. It is regarded that* indigo naturalis* contains two main active ingredients, indirubin and indigo. Indigo is very unstable and easy to be reduced to indigo white [23]. Therefore, indirubin was taken to proceed with the further study.

In this research, geometries and harmonic vibrational frequencies of the As_4_S_4_, indirubin, and complex of [As_4_S_4_^…^Indirubin] that being formed by the former two compounds were fully optimized by using Hartree-Fock (HF) [24] and density functional theory (DFT) [25] methods, where the DFT method using Beck's three-parameter nonlocal exchange functional along with the Lee-Yang-Parr nonlocal correlation functional (B3LYP) [26, 27] and the wB97XD functional including a long-range correction and accounting for dispersive forces from Chai and Head-Gordon[28]. The standard 6-31G*∗* basis set of double-zeta contraction quality plus six-d-like polarization functions [29] and the 6-31+G*∗* basis set with diffuse function were performed in the present study. In addition, the correction of the basis set superposition error (BSSE) by the counterpoise method [30] of complex [As_4_S_4_^…^Indirubin] has been investigated at the wB97XD /6-31+G*∗* level of theory. Throughout this paper, bond lengths are given in Å angstroms, total energies and BSSE correction in \, zero-point energies, and binding energies in kcal/mol. All calculations are performed with Gaussian 09 program package [31] and the Gaussian View program.

**Figure 6:**
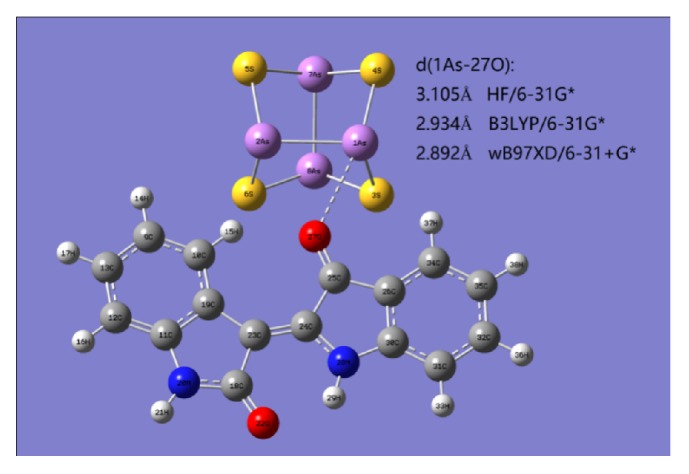
The geometry of complex of [As_4_S_4_^…^Indirubin] at the HF/6-31G*∗*, B3LYP/6-31G*∗*, and wB97XD/6-31+G*∗* levels of theory. (The above cradle type of actatomic ring is the geometry of *α*-As_4_S_4_; purple spheres represent As atoms and yellow spheres represent S atoms. The lower rings represent the geometry of indirubin; red spheres represent O atoms and blue spheres represent N atoms.)

Total energies with zero-point energy (ZPE) of As_4_S_4_, indirubin and complex of [As_4_S_4_^…^Indirubin], and BSSE of complex [As_4_S_4_^…^Indirubin] at the HF/6-31G*∗*, B3LYP/6-31G*∗*, and wB97XD/6-31+G*∗* levels of theory are shown in Table 4. The geometry and harmonic vibrational frequency analyses show that the complex of [As_4_S_4_^…^Indirubin] is a local minimum with all real frequencies. As shown in Figure 6, the present calculated As-O distance d(1As-27O) is 3.105, 2.934, and 2.892 Å at the HF/6-31G*∗*, B3LYP/6-31G*∗*, and wB97XD/6-31+G*∗* levels of theory, respectively, which is larger than the sum of the covalent radii of the As and O atoms (1.84Å), but shorter than the sum of the van der Waals radii of the As and O atoms (3.37Å), indicating that the *α*-As_4_S_4_ and indirubin (C_16_H_10_N_2_O_2_) may form a complex of [As_4_S_4_^…^Indirubin] by the noncovalent interaction. The predicted binding energy with ZPE and BSSE corrections is 0.3, 0.3, and -5.8 kcal/mol at the HF/6-31G*∗*, B3LYP/6-31G*∗*, and wB97XD/6-31+G*∗* levels of theory, respectively, listed in Table 5. Considering the importance of using larger basis sets as well as adequately correlated methods to compute properties of noncovalent interactions, the predicted binding energy of -5.8 kcal/mol at the wB97XD/6-31+G*∗* level of theory is more reasonable than the other two.

Computational study shows that As_4_S_4_ and indirubin form a noncovalent interaction through linking As and O atoms. It is predicted that the noncovalent interaction between As_4_S_4_ and indirubin raises the solubility and improves the releasing degree into bloodstream of As_4_S_4_ owing to the organic characteristics of indirubin.

## 5. Conclusion

The phenomenon which occurred in the pharmacokinetic study illustrated that the combination medication of realgar and* indigo naturalis* enhanced the arsenic AUC in blood. This represented the fact that the arsenic of combination group had higher bioavailability in mice bodies. Realgar had higher C_max_, while it had shorter t_half_ and MRT, which meant realgar reached the peak concentration in a short time and it was eliminated in a relatively short period. Multiple oral administration study future illustrated that the combination medication of realgar and* indigo naturalis* enhanced the arsenic concentration in blood. The MDCK-MDR1 based permeability experiments verified the transport rate and penetration amount of arsenic increased when two drugs were used together in both A-B and B-A pathways.* Indigo naturalis* enhanced the arsenic penetration amount and permeability rate. The MDCK-MDR1 based permeability results were consistent with the pharmacokinetic study. Cytotoxicity study proved that the combination therapy efficiently decreased the survival of K562 cell line, while the cell viability was not decreased during the single medication at the same single concentration. These experiments suggested that the combination administration of realgar and* indigo naturalis* could produce synergistic action. An apoptotic study found that R and RI reduce the viability of K562 cells through inducing cells apoptosis and the combination medication of realgar and* indigo naturalis* increases the apoptotic effect of K562 cells. A theoretical result was provided by the computational work that the formation of the complex [As_4_S_4_^…^Indirubin] which involves noncovalent interaction might be another reason that promotes the arsenic concentration.

Realgar and* indigo naturalis* are both traditional oral drugs for treating various types of leukemia, commonly being combination medication. Starting with the combinatorial modes in traditional medicines to find mechanisms has advantages of sufficient clinical experiences accumulated in hundreds of years [32]. United applications of pharmacokinetics, cell transport, and computational quantum chemistry explore the drug-drug interactions, possibly offering a novel line for multicomponents analysis [33].

## Figures and Tables

**Table 1:** Microwave digestion system setting steps.

Time	Temperature	Power
0~3min	0→120°C	1600 W
3~6 min	120°C	1600 W
6~9 min	120°C→150°C	1600 W
9~12 min	150°C	1600 W
12~17 min	50°C→180°C	1600 W
17~27 min	180°C	1600 W
27~60 min	180°C→25°C	0 W

**Table 2:** Pharmacokinetic parameters of arsenic after oral administration of realgar and realgar-*indigo naturalis*.

Parameters	Unit	realgar	realgar+ *indigo naturalis*
T_max_	h	0.75	0.75
C_max_	*μ*g L^−1^	2472.41	1681.32
AUC_(0-24)_	h *μ*g L^−1^	18992.00	24057.60
AUC_(0-∞)_	h *μ*g L^−1^	22039.20	30343.00
t_half_	h	8.58	9.91
MRT	h	12.97	15.57
C_max_ R/RI		1.47	
AUC_(0-24)_ R/RI		0.79	
AUC_(0-∞)_ R/RI		0.73	

**Table 3:** Apparent permeability coefficients of transported arsenic in R and RI across MDCK-MDR1 cells.

Group	Papp (×10^−6^ cm/s)		ER
	A-B	B-A	
R	7.63±0.0100	5.17±0.0066	0.68
RI	10.45±0.0136	6.53±0.0085	0.63

**Table 4:** Total energies [a.u.] with ZPE [kcal/mol] in parentheses of As_4_S_4_, indirubin, complex of [As_4_S_4_^…^Indirubin], and BSSE of complex [As_4_S_4_^…^Indirubin] at the HF/6-31G*∗*, B3LYP/6-31G*∗*, and wB97XD/6-31+G*∗* levels of theory.

Structures	HF/6-31G*∗*	B3LYP/6-31G*∗*	wP97DX/6-31+G*∗*
As_4_S_4_ (*D*_2*d*_)	-10518.24526 (8.0)	-10528.05290 (7.0)	-10528.08202 (7.4)
Indirubin C_16_H_10_N_2_O_2_ (*C*_1_)	-870.37812 (152.6)	-875.71055 (142.1)	-875.44319 (143.8)
complex (*C*_1_)	-11388.64324 (161.5)	-11403.78579 (149.9)	-11403.57410 (152.2)
BSSE energy	0.02180	0.02400	0.04130

**Table 5:** Binding energies (in kcal/mol) with BSSE correction values in parentheses of complex [As_4_S_4_^…^Indirubin] relative to separated As_4_S_4_ and indirubin (C_16_H_10_N_2_O_2_) at the HF/6-31G*∗*, B3LYP/6-31G*∗*, and wB97XD/6-31+G*∗* levels of theory.

Structures	HF/6-31G*∗*	B3LYP/6-31G*∗*	wB97XD/6-31+G*∗*
[As_4_S_4⋯_C_16_H_10_N_2_O_2_]	-13.4 (0.3)	-14.8 (0.3)	-31.7 (-5.8)

The binding energy for the complex was evaluated according to the following definition given in the following equation: E_binding_ = E_complex_- E_AS4S4_- E_indirubin_ + BSSE).

## Data Availability

The data used to support the findings of this study are available from the corresponding author upon request.

## Abbreviations

As: Arsenic
O: Oxide
As_4_S_4_: Tetraarsenic tetrasulfide
As_2_O_3_: Arsenic trioxide
APL: Acute promyelocytic leukemia
MDS: Myelodysplastic syndromes
CML: Chronic myelogenous leukemia
FDA: The US Food and Drug Administration
TCM: Traditional Chinese medicine
ICP-MS: Inductively coupled plasma mass spectrometer
RBCs: Red blood cells
R: Realgar
I: * Indigo naturalis*.
